# Best evidence summary for the rational use of parenteral nutrition in hospitalized cancer patients

**DOI:** 10.3389/fnut.2025.1730398

**Published:** 2026-01-21

**Authors:** Zhengzheng Liu, Beijia Liu, Niannian Weng, Qian Gui, Di Liu, Yuchi Wu, Guiyu Huang, Mingxue Yang, Xiaoli Tang

**Affiliations:** 1 School of Medicine, University of Electronic Science and Technology of China, Chengdu, China; 2 Department of Vascular and Interventional Surgery, Affiliated Cancer Hospital of Chongqing University, Chongqing, China; 3 Department of Arthroplasty and Sports Medicine Ward, The Third People’s Hospital of Chengdu, Chengdu, Sichuan, China; 4 Department of Radiation Oncology, Sichuan Cancer Hospital, Affiliated Cancer Hospital of University of Electronic Science and Technology of China, Chengdu, China; 5 Department of Comprehensive Ward, Sichuan Clinical Research Center for Cancer, Sichuan Cancer Hospital & Institute, Sichuan Cancer Center, University of Electronic Science and Technology of China, Chengdu, China

**Keywords:** cancer, nutrition support, parenteral nutrition, quality management, summary of evidence

## Abstract

**Objective:**

To systematically identify, review, and synthesize the best available evidence on the rational use of parenteral nutrition (PN) in hospitalized adult oncology patients, and to develop a practice-oriented framework encompassing decision-making, prescribing, review, compounding, administration, and monitoring, and quality management.

**Methods:**

PIPOST-based questions were developed to guide the review. Following the “5S” evidence-pyramid model, searches were performed in a top-down manner across system-level resources, guideline repositories, synthesis databases, and primary literature databases, including UpToDate, BMJ Best Practice, NICE, ESPEN, CSPEN, ASPEN, Chinese Certified Dietitian, Cochrane, JBI, PubMed, Embase, Web of Science, CINAHL, CNKI, Wanfang, and SinoMed. Eligible evidence types included clinical decision resources, clinical practice guidelines, systematic reviews and meta-analyses, expert consensus statements, and evidence summaries related to parenteral nutrition for hospitalized adult cancer patients. Study selection, quality appraisal, and data extraction were conducted independently by two trained reviewers, and any disagreements were resolved through discussion or adjudication by a third reviewer. Evidence items were regraded using the JBI pre-grading framework and synthesized thematically. The search covered all databases from their inception to 13 August 2025.

**Results:**

A total of 2,248 records were retrieved. Eighteen documents met the inclusion criteria and were included: one system-level clinical decision resource, four clinical practice guidelines, nine expert consensus statements, and four systematic reviews. From these sources, 46 discrete evidence items were distilled and organized into five domains: individualized nutritional decision-making, PN prescribing and review, PN preparation and compounding, PN administration and monitoring, safety assessment and quality management.

**Conclusion:**

Parenteral nutrition for hospitalized cancer patients should be implemented within a multidisciplinary Nutrition Support Team (NST) framework and embedded within institutional quality management systems. Structured, individualized care plans should be developed based on the best available evidence. Given variability in institutional resources, staff competencies, and evidence across tumor subgroups, key quality indicators should be specified, and routine audits should be conducted during local implementation. The ultimate goal is to improve nutritional status, clinical outcomes, and the efficiency of healthcare resource utilization among hospitalized cancer patients.

## 1 Introduction

Malnutrition is a common clinical problem among patients with malignant tumors, with reported prevalence rates of approximately 30–70% (1), and approximately 20% of cancer deaths have been attributed directly to malnutrition (2). The determinants of cancer-related malnutrition include tumor-related and treatment-related factors (e.g., reduced oral intake, metabolic alterations, and adverse effects of therapy), and malnutrition, in turn, further exacerbates patient morbidity. Studies have shown that malnutrition substantially reduces treatment tolerance, increases treatment interruptions and complication rates, prolongs hospital stay, impairs quality of life, and ultimately heightens mortality (3–6). Moreover, malnutrition has been closely associated with psychological morbidity. One study reported that hospitalized cancer patients with malnutrition had an increased risk of anxiety (odds ratio 1.98; 95% CI 1.01–3.98; *p* = 0.049) and an increased risk of depression (odds ratio 6.29; 95% CI 1.73–20.47; *p* = 0.005) (7). Effective management of nutritional problems is therefore indispensable to comprehensive oncology care.

Clinical guidelines recommend a stepwise approach to nutritional support. For patients at nutritional risk or who are malnourished, enteral nutrition (EN) is preferred when feasible; parenteral nutrition should be used when EN and oral intake are insufficient to meet requirements. When EN is expected to provide less than 50–60% of energy and protein targets, PN is generally initiated within 3–7 days to maintain energy and nitrogen balance (8). Nutrients are delivered intravenously by PN, thereby bypassing the gastrointestinal tract, and PN can preserve energy stores and body mass when enteral feeding is not possible. PN is particularly useful for patients with severe intestinal dysfunction—such as bowel obstruction, radiation enteritis, short-bowel syndrome, or chylothorax—when EN is not feasible (9). Evidence indicates that PN can maintain energy and nitrogen balance in patients undergoing surgery or chemoradiotherapy and may reduce infection rates and postoperative complications in selected settings (10, 11). In addition, PN may improve tolerance to oncologic therapies and treatment continuity, which can translate into improved survival and quality of life for some patient groups (12).

However, PN is not without risks. Because PN is administered intravenously and comprises complex formulations, careful prescribing, aseptic compounding, and meticulous administration management are required. Inadequate control at any stage can result in formulation instability, precipitation, or contamination, and may lead to phlebitis, catheter-related bloodstream infection, dysglycemia, and electrolyte disturbances (13, 14). When standard operating procedures are strictly followed, PN does not increase the incidence of infectious complications compared with EN (15, 16). Therefore, the implementation of standardized, end-to-end PN protocols tailored to hospitalized oncology patients is necessary to ensure nutritional efficacy, minimize safety and complication risks, and improve patient adherence and clinical outcomes.

Currently, evidence regarding PN use in hospitalized cancer patients is fragmented and lacks systematic synthesis, and existing guidelines provide general recommendations but rarely offer a focused, full-process evidence summary encompassing prescription review, compounding, infusion monitoring, and quality management. To address this gap, a systematic, evidence-based search and synthesis of high-quality literature was performed to develop a practical, evidence-informed framework for the rational use of PN in hospitalized cancer patients, with the objective of providing clinical healthcare professionals with clear, actionable guidance to optimize nutritional support and patient outcomes.

## 2 Materials and methods

### 2.1 Establishment of evidence-based questions

To guide the evidence summarization, specific questions were established based on the PIPOST model (17).

P (Population): Hospitalized adult cancer patients (≥18 years).

I (Intervention): Administration of parenteral nutrition support.

P (Professionals applying evidence): Clinicians, nutrition support dietitians, pharmacists, nurses, and other members of the multidisciplinary team.

O (Outcomes): Incidence of complications, laboratory test indicators, nutritional status, and quality-management indicators.

S (Setting): Oncology specialty hospitals, oncology wards of general hospitals, and parenteral nutrition compounding centers.

T (Type of evidence): Thematic evidence summaries (including clinical decision-making, practice recommendations, and evidence summaries), clinical practice guidelines, systematic reviews, expert consensus statements, and expert opinions.

Definition note: Nutrition support dietitians are defined according to ASPEN as professionals who perform individualized nutrition assessment, develop and implement nutrition care plans, monitor patient responses to nutrition therapy, and coordinate transitional or discontinuation plans for nutrition support (18, 19).

### 2.2 Inclusion and exclusion criteria

Inclusion criteria:

1. Studies involving hospitalized cancer patients aged ≥18 years.
2. Research addressing parenteral nutrition support.
3. Types of evidence included: the latest guidelines, evidence summaries, expert consensus, clinical decision-making, recommended practices, systematic reviews, and meta-analyses.
4. Language of publication: Chinese or English.

Exclusion criteria:

1. Guideline interpretations, translated versions, or duplicate publications.
2. Inaccessible full texts or incomplete literature information.
3. Studies that fail to meet quality-assessment standards.
4. Conference abstracts, discussion papers, or other unpublished materials.

### 2.3 Search strategies

Using the “5S” evidence-pyramid model (20), the terms “cancer,” “nutrition,” and related keywords were used to search relevant Chinese- and English-language guideline websites, professional society websites, and the UpToDate in a top-down manner. Systems-level resources searched included UpToDate and BMJ Best Practice. Synthesized-summary and guideline sources searched included the National Guideline Clearinghouse (NGC), the Scottish Intercollegiate Guidelines Network (SIGN), the National Institute for Health and Care Excellence (NICE), the Guidelines International Network (GIN), the Registered Nurses’ Association of Ontario (RNAO), Medlive, the European Society for Clinical Nutrition and Metabolism (ESPEN), the American Society for Parenteral and Enteral Nutrition (ASPEN), the Chinese Society for Parenteral and Enteral Nutrition (CSPEN), and the Chinese Certified Dietitian. Synthesis databases searched included the Cochrane Library and the Joanna Briggs Institute (JBI) Library. Primary study databases searched included PubMed, Embase, Web of Science, and CINAHL. Chinese databases searched included China National Knowledge Infrastructure (CNKI), Wanfang Data, VIP, and the China Biology Medicine Database (CBM). The search period extended from each database’s inception to 13 August 2025. The PubMed search strategy is detailed in the Supplementary material S1.

### 2.4 Literature quality assessment

To ensure the reliability and interpretability of the evidence synthesis, quality-appraisal tools appropriate to each type of included literature were applied. The included evidence types comprised clinical decision resources, clinical practice guidelines, expert consensus statements, and systematic reviews. The appraisal methods were as follows: (1) Clinical decision resources: Because no universally accepted instrument exists for appraising system-level clinical decision–support content, items obtained from authoritative clinical decision platforms or system databases were provisionally classified as high-quality clinical decision evidence, and source provenance and last-update date were recorded (21). (2) Clinical practice guidelines: The Appraisal of Guidelines for Research and Evaluation (AGREE II) (Table 1) (22) instrument was used. AGREE II comprises six domains and 23 items; each item is scored from 1 to 7 (1 = strongly disagree; 7 = strongly agree). Domain scores were calculated and used to generate an overall assessment. Guidelines were classified as grade A (all six domain scores ≥60%), grade B (≥3 domains scoring 30–60%), or grade C (>3 domains scoring <30%). (3) Expert consensus (Supplementary Table S2) and systematic reviews (Supplementary Table S3) were evaluated using the criteria of the Authentic Assessment Tool (2016) (23) from the Australian JBI Center for Evidence-Based Health Care.

**Table 1:** Guide quality evaluation results.

Guideline	Percentage standardization by area (%)						≥60% field number (n)	≤30% field number (n)	Recommended level
	Scope and purpose	Stakeholder involvement	Rigour of development	Clarity of presentation	Applicability	Editorial independence			
National Institute for Health and Care Excellence (25)	100	75.93	75	99.07	77.08	79.17	6	0	A
Chinese Society of Parenteral and Enteral Nutrition (CSPEN) (24)	98.15	79.63	93.06	92.59	82.64	91.67	6	0	A
Muscaritoli et al. (26)	99.07	85.19	82.64	97.22	68.75	87.5	6	0	A
Arends et al. (27)	99.07	82.41	92.71	97.22	77.08	91.67	6	0	A

Two reviewers trained in evidence-based methods independently and blind to each other’s assessments appraised each included document, and item-level scores and principal methodological limitations were recorded. Disagreements were resolved through discussion; if consensus was not reached, a third reviewer with methodological expertise adjudicated. All final quality assessments and the rationale for judgments were documented.

### 2.5 Evidence summary and grading

Two reviewers trained in evidence-based methods independently extracted and summarized evidence from the included documents. The following rules guided extraction and synthesis: retain original wording for evidence items that are independently stated; split multi-element statements into discrete items; when multiple sources report essentially the same finding, adopt the clearest and most concise wording; merge complementary findings according to logical relations; for conflicting evidence, trace back to sources and prioritize the evidence with higher methodological quality and more recent publication date, documenting the rationale for selection. Original citation numbers are retained for traceability. Evidence was regraded using the Joanna Briggs Institute (JBI) Levels of Evidence. Discrepancies in extraction or grading were resolved by discussion between the two reviewers, with a third reviewer adjudicating unresolved disagreements.

## 3 Results

### 3.1 Search results

**Figure 1:**
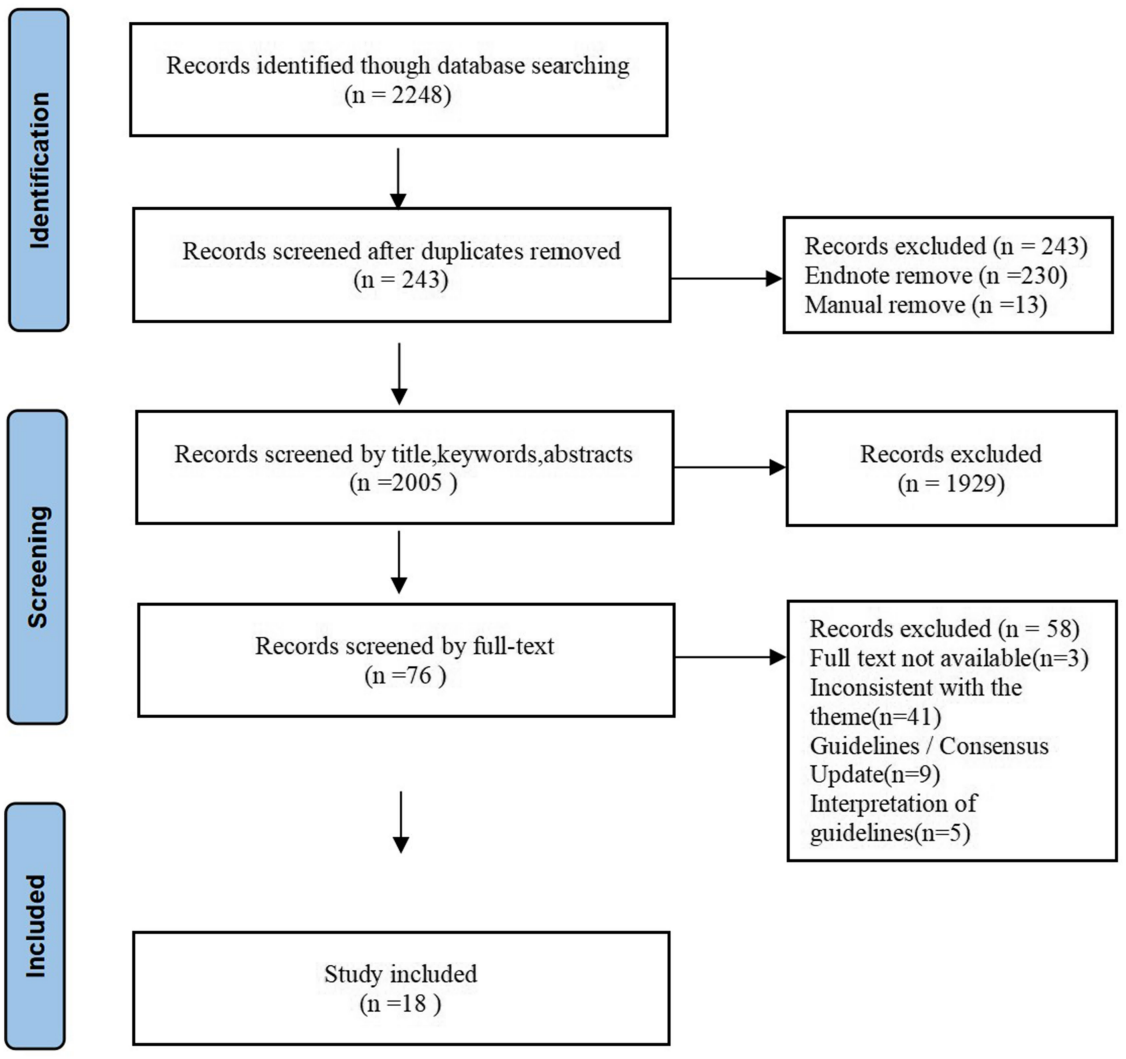
PRISMA flow diagram of literature selection.

A total of 2,248 records were identified through database and website searches. After deduplication with EndNote version 20.0, 2,005 unique records remained. Two reviewers (BL and NW) independently screened titles and abstracts, excluding 1,929 records judged irrelevant and leaving 76 articles for full-text assessment. Upon full-text review, 58 articles were excluded: 44 were not relevant to the topic, 9 were updated versions of existing guidelines or consensus statements, and 5 were guideline interpretations or commentaries. A total of 18 articles met the inclusion criteria and were retained for evidence extraction and synthesis. These comprised one systems-level clinical decision resource, four clinical practice guidelines, nine expert consensus statements, and four systematic reviews. The literature selection process is shown in the PRISMA flow diagram (Figure 1).

The main characteristics of the included studies are summarized in Table 2.

**Table 2:** General information of included articles (*n* = 18).

Included articles	Published year	Source	Type	Theme
David (57)	2025	Up To Date	Clinical decision	Nutrition support in critically ill adults: parenteral nutrition indications, formulation, infusion, and monitoring.
National Institute for Health and Care Excellence (25)	2017	NICE	Guidelines	Comprehensive guidance on screening, indications, implementation, and monitoring for oral, enteral, and parenteral nutrition in adults.
Chinese Society of Parenteral and Enteral Nutrition (CSPEN) (24)	2023	Medlive	Guidelines	Clinical application of parenteral and enteral nutrition in adults: screening, assessment, diagnosis, and multidisciplinary management.
Muscaritoli et al. (26)	2021	ESPEN	Guidelines	Practical recommendations for nutritional screening, assessment, and tailored interventions across cancer patient subgroups.
Arends et al. (27)	2017	ESPEN	Guidelines	Evidence-based guidance on nutrition care in cancer patients, including energy/protein targets and escalation principles.
Chinese Society of Nutritional Oncology, Chinese Society for Parenteral and Enteral Nutrition (8)	2021	Medlive	Expert consensus	Safety management of parenteral nutrition: nutritional diagnosis, indications, prescription, preparation, infusion routes, and complication prevention.
Chinese Medical Association of Parenteral and Enteral Nutrition Nursing Group (28)	2022	Medlive	Expert consensus	Safe infusion of parenteral nutrition: multidisciplinary roles, operational specifications and complication management.
Guangdong Pharmaceutical Association (29)	2022	Medlive	Expert consensus	Clinical pharmacy practice for parenteral/enteral nutrition: screening, assessment, prescription review, and drug–nutrition interaction management.
Zhao et al. (30)	2018	Medlive	Expert consensus	Standardization of PN solution compounding: compounding procedures, environment, personnel, and labeling.
Chinese Society of Nutritional Oncology, Chinese Society for Parenteral and Enteral Nutrition (31)	2020	Medlive	Expert consensus	Refeeding syndrome in cancer patients: definitions, pathogenesis, risk factors, prevention and treatment.
Berger and Pichard (9)	2017	ASPEN	Expert consensus	Criteria and timing for appropriate parenteral nutrition use: feasibility of EN, indications, and initiation timeframes.
Guenter et al. (32)	2018	ASPEN	Expert consensus	Competency framework for PN administration: training, assessment, and safety practices to reduce administration errors.
Guenter et al. (33)	2015	ASPEN	Expert consensus	Competency model for PN prescribing: multidisciplinary prescribing roles, training, and safeguards to prevent errors.
Virizuela et al. (34)	2017	PubMed	Expert consensus	Nutritional screening and PN/HPN indications in cancer patients, with practical energy/protein targets and follow-up considerations.
McCracken et al. (35)	2025	JBI	Systematic review	Determinants of healthcare professionals’ decision-making on PN in advanced cancer, emphasizing MDT and communication factors.
Stidham et al. (36)	2020	PubMed	Systematic review	Impact of Nutrition Support Team oversight on PN appropriateness in hospitalized adults: effects on appropriateness and utilization.
Eriksen et al. (37)	2021	PubMed	Systematic review	Effects of implementing NSTs on in-hospital PN outcomes: catheter infections, mortality, and inappropriate PN use.
Baudolino et al. (38)	2025	PubMed	Systematic review	Psychological impact of vascular access devices in young PN patients: anxiety, depression, body image, and QoL, and implications for psychosocial care.

### 3.2 Results of the quality evaluation of the literature

#### 3.2.1 Quality evaluation of guidelines

Four guidelines were included in this study and were independently appraised by six reviewers; the results of the quality assessment are presented in Table 1. One guideline originated from China (24) and was produced by the Chinese Society of Parenteral and Enteral Nutrition (CSPEN); it provides recommendations for the clinical application of parenteral and enteral nutrition in adult Chinese patients. One guideline originated from the United Kingdom (25) and was issued by the National Institute for Health and Care Excellence (NICE); it provides comprehensive guidance on screening, indications, implementation, and monitoring of oral, enteral, and parenteral nutrition in adults. The remaining two guidelines were issued by the European Society for Clinical Nutrition and Metabolism (ESPEN): one offers practical recommendations for nutritional screening, assessment, and tailored interventions for cancer subgroups (26), while the other is an evidence-based guideline addressing nutrition care in cancer patients (27). All four guidelines were assigned a Grade A recommendation (Table 1). Intraclass correlation coefficient (ICC) analysis was conducted to assess inter-rater agreement; ICC values for the six appraisers’ quality assessments exceeded 0.75 (*p* < 0.05), indicating good consistency among reviewers (Supplementary Table S1).

#### 3.2.2 Quality evaluation of expert consensus

Nine expert consensus documents were included in the quality appraisal: the Chinese Society of Nutritional Oncology, Chinese Society for Parenteral and Enteral Nutrition (8), Chinese Medical Association of Parenteral and Enteral Nutrition Nursing Group (28), Guangdong Pharmaceutical Association (29), Zhao et al. (30), Chinese Society of Nutritional Oncology, Chinese Society for Parenteral and Enteral Nutrition (31), Berger and Pichard (9), Guenter et al. (32), Guenter et al. (33), and Virizuela et al. (34). Five of the included consensus documents were published in Chinese, and four were published in English. Independent, duplicate appraisals were conducted, yielding a high level of inter-rater agreement across items. For every included document, responses to evaluation items ①–⑤ were affirmative (“Yes”). In contrast, item ⑥ (“Were there inconsistencies between the proposed opinions and previous literature?”) was uniformly rated “No” for all documents. Detailed results for each consensus document are presented in Supplementary Table S2.

#### 3.2.3 Quality evaluation of systematic reviews or meta-analyses

Four systematic reviews and/or meta-analyses were included in the quality appraisal: McCracken et al. (35), Stidham et al. (36), Eriksen et al. (37), and Baudolino et al. (38). Overall, the included reviews satisfied most methodological quality items (①–⑧ and ⑩), indicating that research questions, inclusion criteria, search strategies, sources, study-level appraisal criteria, data-extraction safeguards, and synthesis methods were generally appropriate. However, certain recurrent methodological shortcomings were identified: assessment of potential publication bias (item ⑨) was not performed in McCracken et al. (35) and Stidham et al. (36); the independence of the quality assessment (item ⑥) was unclear or not reported in Stidham et al. (36) and Eriksen et al. (37); and McCracken et al. (35) did not provide explicit recommendations for future research (item ⑪). By contrast, Baudolino et al. (38) met all the evaluated criteria. The detailed item-level judgements for each review are reported in Supplementary Table S3.

### 3.3 Summary of evidence

After extraction, comprehensive synthesis, and analysis of evidence regarding the rational use of parenteral nutrition in hospitalized oncology patients, five key domains were identified: individualized nutritional decision-making; PN prescription and review; PN preparation and compounding; PN infusion and monitoring; and safety assessment and quality management. In total, 46 evidence items were identified (Table 3).

**Table 3:** Best evidence summary for the rational use of parenteral nutrition in hospitalized cancer patients.

Evidence topic	Evidence description	Level
Individualized nutritional decision-making	1. All hospitalized patients should undergo nutritional risk screening upon admission, and outpatients should be screened at their initial visit (24, 25).	4a
	2. Cancer patients should commence screening at diagnosis and be reassessed periodically based on clinical stability (26, 27, 34).	5b
	3. Common tools include the Nutrition Risk Screening 2002 (NRS-2002), the Malnutrition Universal Screening Tool (MUST), the Malnutrition Screening Tool (MST), and the Mini Nutritional Assessment–Short Form (MNA-SF). Critically ill patients are recommended to be assessed using the NUTRIC score; perioperative patients may be assessed with PONS; oncology patients may be assessed with the PG-SGA (24, 25, 27–29).	5b
	4. Patients identified as being at nutritional risk or as malnourished should undergo a comprehensive assessment by a Nutrition Support Team or an experienced clinical practitioner (24, 29).	5b
	5. The Global Leadership Initiative on Malnutrition (GLIM) criteria should be applied as the diagnostic standard for malnutrition in cancer patients (24, 29).	3c
	6. Parenteral nutrition should be considered when oral or enteral nutrition is insufficient, unsafe, or infeasible; PN should be prioritized for conditions such as intestinal failure, short bowel syndrome, intestinal ischemia, and high-output fistula (8, 9, 25, 26).	1c
	7. In well-nourished, clinically stable patients, PN may be initiated if oral or enteral nutrition is expected to be unable to be provided for ≥7 days; in patients at high nutritional risk or already malnourished, PN should be considered within 3–5 (or 3–7) days (8, 9).	3c
	8. Supplemental parenteral nutrition (SPN) should be considered when enteral nutrition (EN) cannot meet ≥50–60% of prescribed energy or protein targets (8, 24, 34).	1c
	9. The initiation of PN in advanced or palliative patients requires a comprehensive assessment of expected survival, functional status, and quality of life, and should involve shared decision-making with the patient and family (9, 26, 27, 34, 35).	5b
PN prescription and review	1. Energy: 25–30 kcal/kg/day is recommended for adult oncology patients; individualization is advised in special circumstances (24, 26, 27, 34).	5b
	2. Protein: 1.2–1.5 g/kg/day is recommended; it may be increased to 2 g/kg/day in hypercatabolic states or specific indications, taking renal and hepatic function into account (24, 26, 27, 34).	5b
	3. Excessively high carbohydrate-to-lipid ratios should be avoided in acute or critical phases (24, 29).	1c
	4. Lipid emulsions should be selected on the basis of metabolic, immunological, and hepatic status, and on the combined composition of different fat sources (8, 24).	5b
	5. Vitamins and minerals should approximate recommended dietary allowances (RDA); empiric high-dose supplementation should be avoided unless indicated (24, 26, 27).	1a
	6. Electrolyte provision is generally based on the Daily Dietary Reference Intake (DRI) and adjusted according to serum electrolyte levels (29).	5b
	7. PN support for cancer patients with prolonged periods of low intake (e.g., dysphagia, mucosal damage from radiotherapy) should be initiated gradually, providing 40–50% of predicted energy needs for the first 24–48 h, and then progressively increased according to tolerance and nutritional status (25–27).	5b
	8. Prescription order should verify indications and contraindications; component selection (fish oil, Gln, vitamins, etc.); dose rationality; route; incompatibilities; drug–nutrition interactions; and container/material compatibility (8, 29).	5b
	9. Joint order review should be conducted by a qualified multidisciplinary Nutrition Support Team comprising clinicians, dietitians, clinical pharmacists, nurse specialists, and, if necessary, ancillary staff such as rehabilitation therapists and laboratory or microbiology personnel (8, 9, 24, 25, 32, 33, 36–38).	3a
	10. Barcode verification, double-checking, independent secondary checks (including pump settings), and traceable labeling for prescription, compounding, and infusion (ward, patient name, components, expiry, etc.) should be implemented (28, 32).	5b
PN preparation and compounding	1. PN should be compounded centrally in an Intravenous Admixture Service (IVAS) (28, 30).	5a
	2. Manual compounding must be performed in a Class B (ISO5) laminar-flow hood or equivalent USP Chapter <797>−compliant sterile compounding environment, including appropriate primary engineering controls and buffer-area requirements, to ensure adequate environmental cleanliness and microbiological monitoring (28, 30).	5a
	3. Preparation personnel must demonstrate competence in aseptic technique, undergo pre-employment health examinations, receive continuing education, and be subject to regular assessment at least annually (30).	5b
	4. Parenteral nutrition admixtures should not be routinely used as carriers for non-nutritive drugs. If other medications must be added, explicit compatibility and stability evaluations and pharmacist review are required (8, 29, 32).	5b
	5. The manual compounding sequence must be followed strictly: add phosphates to the amino-acid or glucose solution first; add other electrolytes and trace elements into the glucose or amino-acid solution; dissolve fat-soluble and water-soluble vitamins separately before adding them to the lipid emulsion or glucose; finally, combine all components into the infusion bag in a single operation with gentle mixing, and perform a visual inspection for abnormalities (29, 30).	5b
PN infusion and monitoring	1. Central venous access (CVAD) should be preferred for PN intended to continue for multiple days or for hyperosmolar formulations (≥900 mOsm/L); peripheral venous access (PVC/PPN) should be limited to short-term use (generally ≤10–14 days) or hypotonic formulations (≤900 mOsm/L) (8, 9, 25, 28, 57).	5b
	2. For short-term use (<30 days), a peripherally inserted central catheter (PICC) or a short-term central venous catheter (CVC) may be used; for long-term use (≥30 days), a tunneled TCVC or an implantable PORT should be preferred, particularly for oncology patients requiring prolonged or repeated vascular access. In oncology practice, PN is commonly administered via PICC when peripheral insertion of a central catheter is selected; chemotherapy patients commonly receive PN via implantable venous ports (PORT) (8, 9, 24, 34, 57).	5b
	3. When selecting catheters, the principle of using the minimum number of lumens necessary should be followed (9, 24, 57).	3c
	4. Parenteral nutrition mixtures, including total nutrient admixtures (TNA), require administration through a single 1.2-micron in-line filter. This same 1.2-micron filter should also be used for dextrose–amino acid admixtures and lipid injectable emulsions. For TNAs, the filter should be placed near the catheter hub, while for separate dextrose–amino acid and lipid emulsions administered together, the filter should be positioned below the Y-site (28, 30, 32, 58).	5b
	5. Administration sets (tubing and filters) should be changed every 24 h; when lipids are infused alone, tubing and filters should be changed every 12 h (or according to product instructions) (28, 30, 32).	5b
	6. Infusion devices should be replaced each time nutrition containers are changed. If device integrity is compromised or contamination is suspected, the device should be replaced immediately (28, 30, 32).	5b
	7. Continuous infusion is preferred for critically ill or acutely ill patients; peripheral, home, or intermittent settings may employ intermittent or cyclical infusion (24, 25, 28, 32).	5b
	8. Initial infusion generally provides 50% of the daily target on day 1; if tolerated without severe hyperglycemia or electrolyte disturbances, the infusion may be advanced to the target rate on day 2 (25, 28, 57).	5b
	9. Typical continuous infusion rates range from 40 to 150 mL/h; intermittent infusion rates may reach 200–300 mL/h, depending on formulation and patient tolerance (28).	5a
	10. Qualified clinical personnel should regularly reassess indications, vascular access, risks, and treatment goals (24, 25, 57).	5b
	11. Serum electrolytes (Na, K, Cl, Ca, Mg, PO₄) and blood glucose should be measured daily until stability is achieved (25, 28, 57).	5b
	12. Liver function tests (ALT, AST) and bilirubin should be performed at least weekly during the initial weeks, with increased frequency if abnormalities are detected (57).	5b
	13. Triglycerides (TG) should be measured within the first 2–3 days, then weekly until stability is achieved; thereafter, monitoring may be extended to monthly, and monitoring frequency should be increased if TG levels are elevated (28, 57).	5b
	14. Trace elements (including iron) should be assessed routinely every 3 months in non-ICU patients, and earlier if deficiency is suspected or following formula changes (57).	5b
	15. Daily or regular recording of fluid balance, body weight, vital signs, infusion reactions (fever, allergy, phlebitis, signs of catheter-related infection), and ECG monitoring for high-risk refeeding patients should be performed; interventions should be adjusted immediately if metabolic or clinical abnormalities occur (25, 32, 57).	5b
	16. Daily or regular evaluation of the continued need for PN should be conducted; when oral intake or enteral nutrition meets ≥50–75% of energy and protein requirements and gastrointestinal function recovers, PN should be weaned using a standardized protocol (9, 25).	5b
Safety assessment and quality management	1. Catheter-related bloodstream infection (CRBSI), intestinal failure-associated liver disease (IFALD), refeeding syndrome (RFS), infusion-interruption rate, infusion-failure rate, and delays or interruptions caused by formula changes should be used as operational safety and process-reliability indicators (9, 28, 31, 32).	5b
	2. Patient-experience and quality-of-life/psychological-impact indicators should be incorporated (38).	3b
	3. PN order review forms, PN order checklists, and compounding verification forms that include indication, formula components, compatibility assessments, and prescriber and reviewer signatures, etc., should be established and used (29, 30, 32, 33).	5b
	4. Infusion records and nursing checklists (including patient ID, formula batch number, start and end times, pump settings, double-check records, filter changes and change times, etc.) should be established and retained as routine quality-control archives (28, 30, 32, 33).	5b
	5. Technological measures, such as smart pumps and barcode systems, should be introduced to reduce infusion errors (32, 33).	5b
	6. Continuous quality improvement (QI) processes should be established, including routine clinical audits, PDSA (plan–do–study–act) cycles, and drug-use evaluations to track PN appropriateness, complication rates, and the effectiveness of improvement measures (9, 28, 32, 33).	5b

## 4 Discussion

### 4.1 Initiation of PN based on individualized assessment of indications and nutritional needs

In hospitalized oncology patients, nutritional screening followed by comprehensive assessment remains essential for determining the appropriate timing of PN (39). Validated tools such as NRS-2002 and PG-SGA should be applied at diagnosis and upon admission, with reassessment triggered by clinical instability. Abnormal screening results require multidisciplinary evaluation of dietary intake, body-composition changes, metabolic markers, and refeeding-syndrome risk, owing to the highly dynamic nature of cancer trajectories. Differences among current guidelines indicate variability in operational thresholds for PN initiation. For example, CSPEN recommends initiating supplemental PN in high-risk patients (e.g., NRS-2002 ≥5 or NUTRIC ≥6) when enteral nutrition fails to provide ≥60% of required energy and protein within 48–72 h (24). ASPEN similarly advises initiating PN within 3–5 days in nutritionally at-risk patients unlikely to achieve sufficient oral intake or EN, while recommending deferring PN in cases of severe metabolic instability until clinical stabilization is achieved (9).

Although a retrospective cohort study suggested that early PN initiation may benefit patients with moderate to severe malnutrition (40), decisions regarding PN in advanced cancer should also incorporate expected survival, functional status, and patient preferences, as emphasized by multiple expert consensus statements (9, 26, 27, 34, 35). These differences reflect variation in both the certainty of the supporting evidence and the clinical priorities across regions. Implementation challenges persist, including limited availability of Nutrition Support Teams (NST), inconsistent training in PG-SGA and GLIM, and inadequate resources to support serial reassessment. These constraints diminish the reliability and generalizability of guideline-based PN initiation. Establishing structured screening–assessment pathways, ensuring NST oversight, and standardizing reassessment intervals may improve the appropriateness and consistency of PN initiation.

### 4.2 Personalized formulation and progressive advancement according to metabolic status

Parenteral nutrition formulations for oncology patients must be individualized to meet metabolic demands while minimizing metabolic burden. Energy targets for adult patients generally range from 25 to 30 kcal/kg/day, and protein requirements typically fall between 1.0 and 1.5 g/kg/day, with higher amounts indicated in hypercatabolic states (27, 41). Appropriate macronutrient distribution and strict control of glucose infusion rates (42) are essential to prevent metabolic complications such as hyperglycemia, while the selection of lipid emulsions (43–45) should reflect metabolic and immunological status and be adjusted in cases of hypertriglyceridemia. Compared with fixed formula patterns, adopting a personalized formulation strategy is critical, incorporating tumor type, disease trajectory, treatment tolerance, organ function, and dynamic metabolic status. This individualized approach highlights the need for stronger empirical evidence, as many current formulation recommendations are grounded in expert consensus rather than high-quality comparative trials. More robust RCTs evaluating formulation components in specific cancer subtypes would substantially strengthen the evidence base.

PN advancement should follow a gradual, risk-stratified escalation strategy, particularly for patients at high risk of refeeding syndrome (RFS), where conservative initiation and intensified metabolic monitoring are required (25–27). Studies have shown that the energy administered on the first day of PN is independently associated with the development of RFS (46, 47). Effective implementation of individualized PN requires coordinated multidisciplinary collaboration. Daily documentation, reassessment of prescribed goals versus actual infusion, and continuous metabolic evaluation are essential to guide timely adjustments. In practice, limited capacity for routine monitoring of triglycerides, electrolytes, and liver function restricts metabolism-guided titration, while insufficient NST staffing and fragmented workflow integration impede effective interdisciplinary collaboration. Establishing standardized procedures under NST supervision, together with risk-stratified advancement strategies, may enhance safety, reduce variability, and support the translation of evidence into clinical practice (32, 36, 37). Optimizing collaboration among multidisciplinary team members and implementing an NST-led prescription review system may further strengthen consistency and promote evidence-based PN formulation.

### 4.3 Aseptic compounding under pharmacist verification to ensure compatibility and safety

The preparation of parenteral nutrition is a high-risk compounding procedure that should be performed within centralized, standardized processes under pharmacist supervision to prevent microbial contamination and physicochemical incompatibilities; any breach of aseptic technique can result in contamination and increase the risk of serious infection (48). Whenever possible, manual compounding should be conducted in an institutional IV drug-admixture service (e.g., Pharmacy Intravenous Admixture Service (PIVAS)) within a Class B (ISO 5) laminar-flow hood or equivalent USP Chapter <797>−compliant sterile compounding environment (30, 49). The central principle is that safe PN preparation depends on robust environmental control, validated aseptic technique, and consistently applied compounding workflows (28). Pharmacist verification is essential, given the complexity of PN electrolyte–nutrient matrices and the high risk of calcium–phosphate precipitation (50–52). In clinical practice, PN admixtures may be used as a vehicle for drug infusion to reduce the patient’s fluid burden (14, 30, 53). However, the multi-component nature of PN makes compatibility unpredictable, and any medication addition must undergo formal compatibility and stability assessment with pharmacist approval (8, 29, 32).

Growing evidence (48, 54) shows that pharmacist-led services improve patient safety and reduce healthcare expenses, and ASPEN recommends pharmacist engagement in nutrition-support safety and quality-improvement programs (55). Pharmacists are positioned to identify root causes of compounding errors, develop targeted corrective strategies, and coordinate interdisciplinary execution. Given these responsibilities, optimizing PN compounding practices requires not only adherence to technical standards but also organizational systems that support pharmacist oversight and team-based coordination. To enhance safety and scalability, institutions should establish standardized compounding Standard Operating Procedures (SOPs), strengthen pharmacist-led prescription review, integrate intelligent verification technologies (e.g., barcode/RFID checks, automated compatibility databases), reinforce NST collaboration, and accelerate PIVAS capacity development (56). The transition toward smart compounding centers that integrate automation, robotics, and Internet-of-Things (IoT) technologies may further reduce error rates and improve operational efficiency, thereby enhancing the overall safety of PN preparation.

### 4.4 Standardized vascular access and infusion protocols to minimize complications

The correct choice of vascular access is a prerequisite for the safe infusion of PN. Although guidelines commonly recommend central venous access (CVAD) for prolonged or hyperosmolar formulations (8, 9, 25, 28, 57), device selection should be individualized by considering anticipated PN duration, lumen requirements, vascular status, and the patient’s oncologic treatment plan. Implantable ports may be advantageous for oncology patients who require both PN and chemotherapy, as they eliminate the need for additional catheter placement (8, 9, 24, 34, 57). When selecting catheters, the principle of using the fewest lumens necessary should be followed (9, 24, 57); however, further research is required to clarify and confirm whether reserving a dedicated PN lumen influences infection risk.

The standardized use of infusion devices forms another essential safeguard for PN safety. ASPEN now recommends a single 1.2-micron filter for total nutrient admixtures (TNA), dextrose–amino acid admixtures, and lipid emulsions (28, 30, 32, 58). Regular replacement of tubing and filters has been shown to reduce catheter-related bloodstream infection (13, 58), which highlights the importance of consistent device-management protocols. Nurses, as the primary providers of PN at the bedside, play a central role throughout the infusion process by maintaining aseptic technique, assessing catheter sites, detecting early signs of infectious or metabolic complications, verifying pump parameters, managing infusion equipment, educating patients, and communicating promptly with pharmacists, dietitians, and physicians (59). This continuous, coordinated oversight is critical for translating technical standards into safe clinical practice.

The mode and rate of PN infusion should be tailored to the patient’s tolerance (24, 25, 28, 32). Continuous infusion is recommended for critically ill or acutely unstable patients, with gradual titration to target rates over 24–48 h (25, 28, 57). Cyclic PN demonstrates biologically plausible hepatoprotective effects in animal studies (60–62); however, high-quality clinical evidence remains limited. Systematic monitoring of electrolytes, triglycerides, and liver function is essential for dynamic adjustment of PN composition and infusion rates (25, 28, 57). To promote consistent implementation, institutions should establish standardized infusion pathways, strengthen interdisciplinary coordination, and adopt smart-pump technologies to reduce manual variation. Structured training in aseptic technique, vascular-access management, complication recognition, and PN infusion protocols is essential for maintaining personnel competency and ensuring adherence to safety standards.

### 4.5 Implementation of safety indicators and continuous quality improvement for rational PN use

The 2024 International PN Safety and Quality Summit Consensus pointed out that PN quality management remains uneven across regulatory and operational levels, with substantial cross-country variation that directly affects patient safety and therapeutic consistency (63). The rational and safe implementation of parenteral nutrition requires integrating objective safety indicators, patient-centered outcomes, technological safeguards, and multidisciplinary governance into a structured continuous quality improvement (CQI) framework (63, 64). Core indicators—including catheter-related bloodstream infection (CRBSI), intestinal failure–associated liver disease (IFALD), refeeding syndrome (RFS), infusion reliability, and metabolic control—provide healthcare organizations with clear benchmarks for evaluating effectiveness and identifying areas requiring improvement (9, 28, 32, 33). In addition, a systematic review (38) reported that PN catheters may provoke anxiety and concerns about body image, underscoring the need to incorporate patient-experience and quality-of-life outcomes, particularly for individuals who rely on long-term PN.

At the operational level, standardized prescription-review tools, infusion checklists, smart pumps, barcode systems, and automated alerts contribute to more consistent and reliable PN delivery (28–30, 32). To strengthen safety oversight, institutions should implement transparent error-reporting systems capable of capturing both adverse events and near-misses, supported by regular NST-led reviews. Routine audits, PDSA cycles, and medication-use evaluations can facilitate root-cause analyses and guide iterative improvement (65–67). Modern digital tools—including automated compatibility checks, real-time dashboards, and electronic PN quality monitoring—may further reduce variability. By embedding PN care within a mature CQI framework that links safety metrics, patient experience, and system-level learning, healthcare organizations can establish a sustainable infrastructure that reduces preventable harm and supports better long-term outcomes.

**Figure 2:**
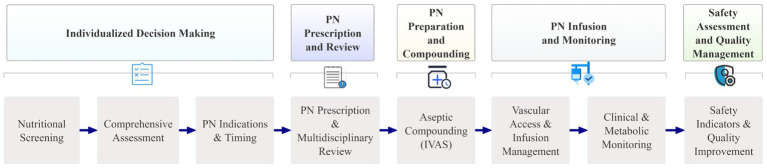
Five-domain framework and stepwise PN clinical pathway.

To further illustrate how the five evidence-based domains operate within an end-to-end clinical workflow, we developed a visual schematic (Figure 2) that links each domain to the sequential clinical steps from nutritional screening to quality improvement. This diagram supports practical implementation by showing how decision-making, prescription, compounding, infusion management, and safety monitoring are integrated across the PN care pathway.

## 5 Conclusion

Parenteral nutrition in oncology care is a dynamic and iterative process. This study synthesizes the best available evidence on PN use in hospitalized oncology patients and, by reorganizing scattered findings into a unified, stepwise framework that can be directly applied in clinical workflows, provides practical guidance for patients whose nutritional and metabolic risks vary throughout the disease course. The framework encompasses five core domains: individualized nutritional screening and assessment; evidence-based indications and timing for PN initiation; personalized formulation with progressive metabolic advancement; aseptic centralized compounding under pharmacist oversight; and standardized vascular-access and infusion management supported by safety indicators and continuous quality improvement.

Translation of these evidence-based components into practice requires careful consideration of institutional resources, staff competencies, and patient characteristics, as these factors may affect the generalizability of the framework. In addition, available evidence for certain tumor subgroups remains limited, particularly regarding optimal PN timing, comparative delivery strategies, and cost-effectiveness. Institutions are therefore encouraged to establish local quality indicators and develop standardized PN safety-monitoring systems that can be integrated into routine audit cycles alongside multidisciplinary NST decision-making. Future work should prioritize prospective implementation studies and multicenter clinical trials evaluating both clinical outcomes and patient-reported outcomes. Overall, this evidence summary offers a clinically deployable framework that supports safer and more standardized PN delivery in hospitalized oncology patients and provides practical reference for related quality-improvement and validation initiatives.

### 5.1 Limitations of the study

Despite providing consolidated evidence on PN use in hospitalized oncology patients, this study has certain limitations. First, restricting the literature search to English and Chinese publications may introduce language bias. Second, the applicability of the synthesized framework may vary across institutions due to differences in resources, staff competencies, PN infrastructure, and oncology care models, potentially contributing to applicability bias. In addition, evidence for specific tumor subgroups and complex clinical scenarios remains limited, which may affect the generalizability of some recommendations. Future updates should expand database coverage, include non-English sources, and incorporate real-world clinical data to enhance external validity.

## Data Availability

The original contributions presented in the study are included in the article/Supplementary material, further inquiries can be directed to the corresponding author.
