# Supplementary material for: Best evidence summary for the rational use of parenteral nutrition in hospitalized cancer patients
Source: Front Nutr. 2026 Jan 21;12:1730398. doi: 10.3389/fnut.2025.1730398 (PMC12867915; doi:10.3389/fnut.2025.1730398)
Supplement: Supplementary file 1 [file Table_1.DOCX]

Supplementary Material

# Supplementary Data

Material S1. PubMed search strategy

Table S1. Results of internal consistency (ICC) test of evaluators

Table S2. Results of the evaluation of the quality of expert consensus.

Table S3. Results of the evaluation of the quality of systematic reviews or meta-analyses

# Supplementary Tables

## Supplementary Material S1: PubMed search strategy

#1 "parenteral nutrition"[MeSH Terms]

#2 "parenteral nutrition"[Title/Abstract] OR "nutrition parenteral"[Title/Abstract] OR "intravenous feeding*"[Title/Abstract] OR "parenteral feeding*"[Title/Abstract] OR "parenteral feedings"[Title/Abstract]

#3 #1 OR #2

#4 "neoplasms"[MeSH Terms]

#5 "neoplasm*"[Title/Abstract] OR "tumor*"[Title/Abstract] OR "neoplasia*"[Title/Abstract] OR "cancer*"[Title/Abstract] OR "malignant neoplasm*"[Title/Abstract] OR "malignanc*"[Title/Abstract] OR "benign neoplasm*"[Title/Abstract]

#6 #4 OR #5

#7 "systematic review"[Publication Type] OR "Meta-Analysis"[Publication Type] OR "practice guideline"[Publication Type] OR "Guideline"[Publication Type] OR "consensus development conference"[Publication Type] OR "consensus development conference, nih"[Publication Type]

#8 #3 AND #6 AND #7

## Table S1: Results of internal consistency (ICC) test of evaluators

| **Guideline** | **ICC** | **95%CI** | ***F*** | ***P*** |
| --- | --- | --- | --- | --- |
| Guideline 1 (25) | 0.823 | (0.682,0.915) | 6.583 | 0.000 |
| Guideline 2 (24) | 0.776 | (0.598,0.892) | 4.498 | 0.000 |
| Guideline 3 (26) | 0.845 | (0.722, 0.925) | 7.134 | 0.000 |
| Guideline 4 (27) | 0.854 | (0.735,0.930) | 8.120 | 0.000 |

## Table S2: Results of the evaluation of the quality of expert consensus

| **Expert consensus** | **①** | **②** | **③** | **④** | **⑤** | **⑥** |
| --- | --- | --- | --- | --- | --- | --- |
| Chinese Society of Nutritional Oncology, Chinese Society for Parenteral and Enteral Nutrition(8) | Yes | Yes | Yes | Yes | Yes | No |
| Chinese Medical Association of Parenteral and Enteral Nutrition Nursing Group(28) | Yes | Yes | Yes | Yes | Yes | No |
| Guangdong Pharmaceutical Association(29) | Yes | Yes | Yes | Yes | Yes | No |
| Pharmacy Cooperative Group, Chinese Society for Parenteral and Enteral Nutrition(30) | Yes | Yes | Yes | Yes | Yes | No |
| Chinese Society of Nutritional Oncology, Chinese Society for Parenteral and Enteral Nutrition(31) | Yes | Yes | Yes | Yes | Yes | No |
| Patricia et al.(9) | Yes | Yes | Yes | Yes | Yes | No |
| Peggi et al.(32) | Yes | Yes | Yes | Yes | Yes | No |
| Peggi et al.(33) | Yes | Yes | Yes | Yes | Yes | No |
| Virizuela J.A. et al.(34) | Yes | Yes | Yes | Yes | Yes | No |
| ① Was the source of the opinions explicitly identified?② Were the opinions derived from influential experts in the field?③ Were the proposed opinions centered on the interests of the population relevant to the research?④ Were the conclusions presented based on the results of analysis, and were the opinions expressed logically?⑤ Were existing literature and evidence cited as references?⑥ Were there inconsistencies between the proposed opinions and previous literature? | | | | | | |

## Table S3: Results of the evaluation of the quality of systematic reviews or meta-analyses

| **Systematic review / Meta-analysis** | **①** | **②** | **③** | **④** | **⑤** | **⑥** | **⑦** | **⑧** | **⑨** | **⑩** | **⑪** |
| --- | --- | --- | --- | --- | --- | --- | --- | --- | --- | --- | --- |
| Jennifer et al.(35) | Yes | Yes | Yes | Yes | Yes | Yes | Yes | Yes | No | Yes | No |
| Morgan et al.(36) | Yes | Yes | Yes | Yes | Yes | Unclear | Yes | Yes | No | Yes | Yes |
| Marcel et al.(37) | Yes | Yes | Yes | Yes | Yes | No | Yes | Yes | Yes | Yes | Yes |
| Mussa et al.(38) | Yes | Yes | Yes | Yes | Yes | Yes | Yes | Yes | Yes | Yes | Yes |
| ① Was the research question clearly defined?② Were the inclusion criteria appropriate for the stated evidence question?③ Was the search strategy appropriate?④ Were the sources of the included research papers appropriate?⑤ Were the criteria used to appraise the quality of included studies appropriate?⑥ Was the quality assessment conducted independently by two or more reviewers?⑦ Were measures taken to minimize errors during data extraction?⑧ Were the methods used to synthesize or combine studies appropriate?⑨ Was potential publication bias assessed?⑩ Were recommendations for policy and/or practice supported by the reported data?⑪ Were appropriate recommendations made for future research directions? | | | | | | | | | | | |
